# Supplementary material for: Immunity against reinfection in pigs following Taenia solium infection and a quantitative dose–response model
Source: Int J Parasitol. Author manuscript; Available in PMC 2026 May 18. (PMC13181579; doi:10.1016/j.ijpara.2025.08.010)
Supplement: sm [file NIHMS2169790-supplement-sm.docx]

**Supplementary information**

**Fig S1** shows the outcomes of the model compared with observed data for pigs infected at 4 weeks of age with 100, 5,000 and 20,000 eggs. Please note that the confidence intervals are confidence intervals for the regression coefficient and not prediction intervals.


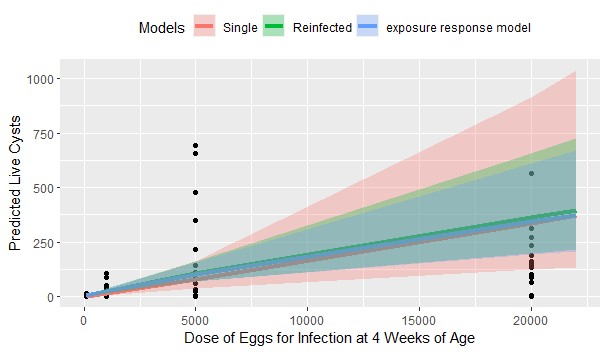


In **Fig S2**, data and predictions from our experiment are compared with data from an earlier systematic review of infection experiments used to feed into a model of the likelihood of infection for different doses (Andrade-Mogrovejo et al., 2022). There is reasonable agreement between our model and prior data. In the prior model, pigs infected with 100 eggs were estimated to have a [62-100%] probability of developing cysts, while in our dataset 11 out of 14 pigs (79%) receiving a first or single infective dose of 100 eggs developed cysts.


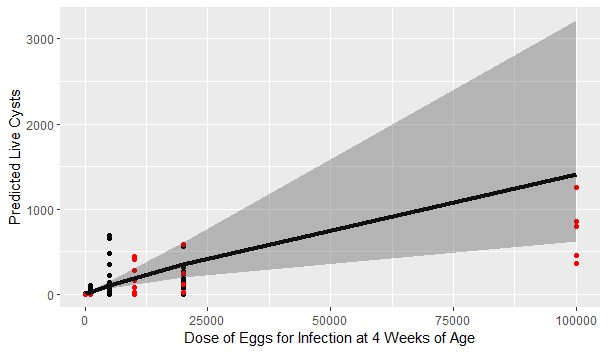


**Fig S3** Individual serological response of each pig throughout the experiment, grouped by infection cluster and dose. Each plot shows the weekly results of two serological tests: LLGP-EITB (bars, right Y-axis) and antigen ELISA (red line, left Y-axis). EITB responses are represented as bars with values ranging from 0 to 7, indicating the number of reactive glycoprotein bands. Antigen ELISA values are shown as a continuous red line. Time in weeks is shown on the X-axis. Each row corresponds to pigs grouped by experimental condition, following the same order as in Table 1: pigs infected once at 4 weeks of age (first four rows), pigs reinfected with 5,000 eggs (rows five to eight), and pigs reinfected with 20,000 eggs (rows nine to twelve). Within each cluster, pigs are further grouped by initial infection dose: 100, 1,000, 5,000, and 20,000 eggs. The final two rows show control pigs infected only once at 16 weeks of age with 5,000 eggs (thirteenth row) and 20,000 eggs (fourteenth row). The first green triangle indicates the time of the first experimental infection, and the second triangle indicates the time of reinfection or necropsy. Serological data for pigs included in the age experiment are reported in the supplementary information of its respective manuscript.


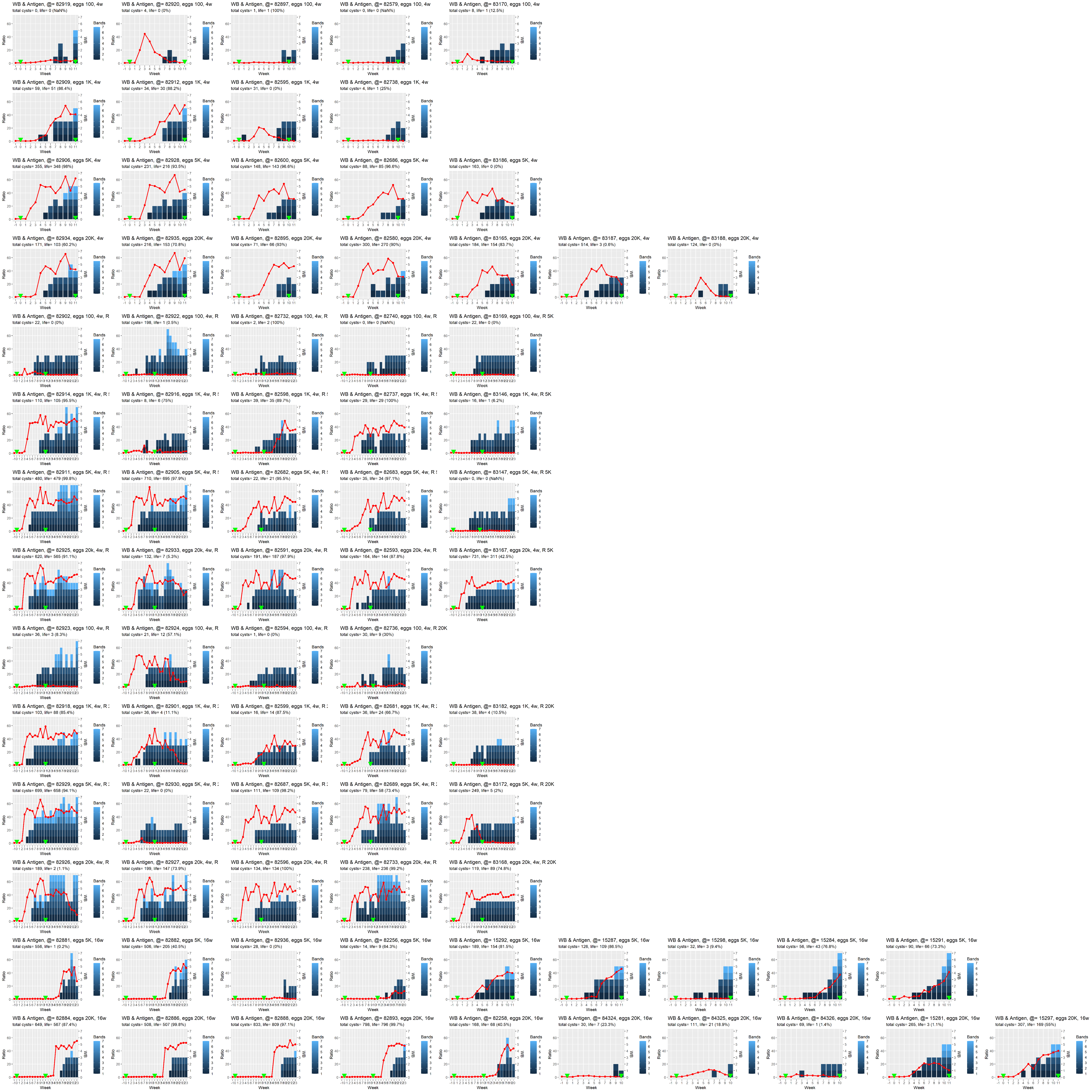


**Table S1.** Number of cysts observed in the brain of pigs by cluster, infection dose, and age at infection. The table shows the number of pigs per group and the number of live, degenerated, and total cysts observed at necropsy, disaggregated by infection age (4 or 16 weeks old). Individual cyst counts per pig are shown when appropriate. For example, “2 (each)” indicates that both animals in the group had 2 cysts each; values like “1, 3 & 34” indicate individual counts per animal within the group. Reinfected pigs were first infected at 4 weeks of age with the dose indicated in the second column, and reinfected at 16 weeks of age with the dose shown in the third column.

| Cluster | Infection at 4 weeks | Infection at 16 weeks | Number of animals | | | Number of cysts in the Brain | | |
| --- | --- | --- | --- | --- | --- | --- | --- | --- |
|  |  |  | Live | Degenerated | Total | Live | Degenerated | Total |
| Pigs infected once | 20,000 | 0 | 2 | 0 | 2 | 2 (each) | 0 | 2 (each) |
| Pigs reinfected with 5000 eggs | 1,000 | 5,000 | 1 | 0 | 1 | 2 | 0 | 2 |
|  | 5,000 | 5,000 | 2 | 0 | 2 | 1 (each) | 0 | 1 (each) |
|  | 20,000 | 5,000 | 4 | 0 | 4 | 1,1, 2 & 9 | 0 | 1,1, 2 & 9 |
| Pigs reinfected with 20000 eggs | 5,000 | 20,000 | 3 | 1 | 3 | 1, 3 & 34 | 6 | 1, 3 & 40 |
|  | 20,000 | 20,000 | 2 | 0 | 2 | 2 & 3 | 0 | 2 & 3 |
| Controls | 0 | 20,000 | 2 | 0 | 2 | 1 & 2 | 0 | 1 & 2 |
| TOTAL |  |  | 16 | 1 | 16 | 67 | 6 | 73 |
